# Peroxiredoxin‐V perturbation in an Alzheimer's disease mouse model

**DOI:** 10.1002/alz70855_106920

**Published:** 2025-12-24

**Authors:** Anant Gupta

**Affiliations:** ^1^ Centre for Brain Research, Banglore, Karnataka, India

## Abstract

**Background:**

Alzheimer's disease (AD), symptomized by cognitive decline and memory loss, is a progressive neurodegenerative disease associated with increased oxidative stress and synaptic dysfunction. Elevated levels of reactive oxygen species (ROS) negatively impact cellular components, including mitochondria. Damaged mitochondria, in turn, contribute to a further increase in ROS levels, triggering apoptotic pathway, and ultimately leading to cell death. Peroxiredoxin‐V (PrxV), one of the oxidoreductive enzymes, is predominantly expressed in neurons and plays a potential role in regulating hydrogen peroxide (H_2_O_2_) levels, thus mitigating oxidative stress. Studies have demonstrated that PrxV prevents Aβ‐induced mitochondrial fragmentation and cell death by reducing oxidative stress. Here, we aimed to examine potential dysregulation of PrxV levels in the AD brain using APP/PS1 transgenic mice at different ages.

**Method:**

To assess oxidative stress, we measured H_2_O_2_ levels in cortical and hippocampal brain tissue lysates using a hydrogen‐peroxide assay kit. We evaluated oxidative stress in primary neuronal culture using CellROX dye. We also analyzed PrxV levels in synaptosomes isolated from wild‐type and APP/PS1 mice using immunoblotting. Additionally, immunocytochemistry (ICC) was performed to examine PrxV levels using a primary antibody against PrxV, while mitochondrial levels were assessed using Mitotracker dye in primary cortical neurons.

**Result:**

In this study, APP/PS1 mice exhibited significantly increased levels of H_2_O_2_ in both hippocampal and cortical tissues compared to age‐matched wild‐type mice. Additionally, PrxV levels were significantly decreased in the synaptosomes of APP/PS1 mice compared to the wild‐type group at one, three, and nine months of age. These immunoblot data were further supported by ICC, as we observed increased CellROX intensity and decreased PrxV intensity in APP/PS1 primary cortical neurons. Mitochondrial intensity was also reduced in APP/PS1 primary neurons compared to wild‐type neurons.

**Conclusion:**

We conclude that perturbation of PrxV levels and mitochondrial dysregulation may contribute to the increased oxidative stress as observed in APP/PS1 brain. Restoring the PrxV levels may mitigate oxidative stress and thus prevent mitochondrial dysregulation and neurodegeneration in AD.

